# Evidence that Natural Selection on Codon Usage in *Drosophila pseudoobscura* Varies Across Codons

**DOI:** 10.1534/g3.114.010488

**Published:** 2014-02-13

**Authors:** Richard M. Kliman

**Affiliations:** Department of Biological Sciences, Cedar Crest College, Allentown, Pennsylvania 18104

**Keywords:** codon bias, natural selection, *Drosophila pseudoobscura*, site frequency spectrum, recombination

## Abstract

Like other species of Drosophila, *Drosophila pseudoobscura* has a distinct bias toward the usage of C- and G-ending codons. Previous studies have indicated that this bias is due, at least in part, to natural selection. Codon bias clearly differs among amino acids (and other codon classes) in Drosophila, which may reflect differences in the intensity of selection on codon usage. Ongoing natural selection on synonymous codon usage should be reflected in the shapes of the site frequency spectra of derived states at polymorphic positions. Specifically, regardless of other demographic effects on the spectrum, it should be shifted toward higher values for changes from less-preferred to more-preferred codons, and toward lower values for the converse. If the intensity of natural selection is increased, shifts in the site frequency spectra should be more pronounced. A total of 33,729 synonymous polymorphic sites on Chromosome 2 in *D. pseudoobscura* were analyzed. Shifts in the site frequency spectra are consistent with differential intensity of natural selection on codon usage, with stronger shifts associated with higher codon bias. The shifts, in general, are greater for polymorphic synonymous sites than for polymorphic intron sites, also consistent with natural selection. However, unlike observations in *D. melanogaster*, codon bias is not reduced in areas of low recombination in *D. pseudoobscura*; the site frequency spectrum signal for selection on codon usage remains strong in these regions. However, diversity is reduced, as expected. It is possible that estimates of low recombination reflect a recent change in recombination rate.

The relative usage of synonymous codons varies among genes within an organism. In some organisms (*e.g.*, humans), this variation may largely reflect base composition variation across the genome (Bernardi *et al.* 1985; Kliman and Bernal 2005). In many organisms, however, natural selection appears to directly influence codon usage, with positive correlations between the levels of codon bias and gene expression that are consistent with selection on transcriptional efficiency and/or fidelity (Chavancy *et al.* 1979; Gouy and Gautier 1982; Ikemura 1985; Akashi 1994; Moriyama and Powell 1997; Kliman and Henry 2005; Plotkin and Kudla 2011). This relationship was first reported for *Drosophila* by Shields *et al.* (1988), and numerous studies using diverse approaches have supported the hypothesis that natural selection influences codon usage in several *Drosophila* species (Kliman and Hey 1993; Akashi and Schaeffer 1997; Carlini and Stephan 2003; Haddrill *et al.* 2011; De Proce *et al.* 2012). Effective weak selection on codon usage requires a sufficient effective population size to overcome the effects of genetic drift [although see Hershberg and Petrov (2008), who point out that selection on codon usage may not be always be weak]. However, evidence has been emerging that natural selection may even be influencing codon usage in humans (Lavner and Kotlar 2005), mammals more generally (Yang and Nielsen 2008), and other vertebrates (Doherty and McInerney 2013).

Among the studies supporting the hypothesis that selection influences codon usage are (1) those that show shifts in the site frequency spectra (SFS) of derived states at synonymous polymorphic sites, such that the SFS is shifted toward higher values for changes to more preferred codons (Akashi and Schaeffer 1997; Kliman 1999; Llopart *et al.* 2008); and (2) those that show significantly reduced codon bias in areas of the genome with very low recombination rates (Kliman and Hey 1993; Hey and Kliman 2002). The latter is consistent with the expectation that natural selection will be less effective in the absence of recombination due to linkage disequilibrium among targets of selection; these are often in repulsion due to independent emergence of selectively favored or disfavored mutations on different copies of a chromosome in a population (Hill and Robertson 1966; Felsenstein 1974; McVean and Charlesworth 2000; Comeron *et al.* 2008). Limited recombination among targets of selection is also predicted to lead to reduced diversity, whether by selective sweeps that wipe out standing variation (Maynard Smith and Haigh 1974; Gillespie 2000) or by background selection against continually arising deleterious mutations that prevent diversity from accumulating in the first place (Charlesworth *et al.* 1993; McVean and Charlesworth 2000). This prediction was most notably confirmed by Begun and Aquadro (1992) in *D. melanogaster* and has been corroborated by subsequent studies in *Drosophila* (Comeron *et al.* 2012) and other organisms [reviewed by Nachman (2002) and Stephan (2010)].

These earlier studies often relied on estimates of recombination rate derived by fitting recombination maps to physical maps, using a variety of line- or curve-fitting approaches. The advent of “next-generation” DNA-sequencing methods has allowed investigators to identify numerous single-nucleotide polymorphisms that can be used in testcrosses to directly estimate recombination rate at a fine scale. Cirulli *et al.* (2007) directly estimated recombination rates across a section of the *D. pseudoobscura* X chromosome and found considerable heterogeneity in recombination rate. Kulathinal *et al.* (2008) showed that estimates of recombination rate at finer scales (*i.e.*, with more densely spaced markers) correlated better with diversity, a finding that suggests that fine-scale recombination rates are more reliable when they can be obtained. McGaugh *et al.* (2012) extended this work to three complete chromosomes, not only confirming heterogeneity, but showing that estimates could be replicated using crosses of different strains. Importantly, McGaugh *et al.* (2012) also sequenced 10 additional *D. pseudoobscura* genomes (along with those of other close relatives) and observed the predicted correlation between recombination rate and diversity. Stevison and Noor (2010) observed a similar relationship between fine-scale recombination rate and diversity in closely related *D. persimilis*.

Although previous polymorphism-based studies on natural selection codon usage in *D. pseudoobscura* have relied on hundreds of variable sites, the Chromosome 2 data from McGaugh *et al.* (2012) provide tens of thousands of variable sites. These data, therefore, allow us to much more thoroughly investigate the effects of natural selection on codon usage in this species. In addition to providing increased statistical power to detect subtle effects, it becomes possible to subdivide data and retain statistical power to test for differential effects. In particular, we investigate whether variation among amino acids in the degree of codon bias can be explained by variation in selection intensity. Furthermore, because of its generally higher recombination rate, analysis with *D. pseudoobscura* provides a valuable contrast to *D. melanogaster*.

As expected, we observe a fairly strong correlation between recombination rate and diversity. Although other composition-biasing influences may be influencing patterns of diversity, most notably G/C-biased gene conversion (Marais *et al.* 2001, 2003; Singh *et al.* 2005), a comparison of SFSs of synonymous and intron sites shows that the SFS shifts are significantly stronger at synonymous sites. Therefore, although selectively neutral influences may be partially responsible for the observed SFS shifts, the data support an influence of natural selection on codon usage. Furthermore, differences among subsets of codons in the SFS shifts are consistent with the differential influence of natural selection. We do not, however, find that selection on codon usage is consistently weaker in areas with lower estimates of recombination rate in *D. pseudoobscura*.

## Materials and Methods

### Data set

Chromosome 2 was recently sequenced in 10 strains of *D. pseudoobscura*, along with one strain of the outgroup *D. lowei*, using Illumina platforms (McGaugh *et al.* 2012) (National Center for Biotechnology Information sequence read archive accession numbers SRA044960.1, SRA044955.2, and SRA044956.1). The reference strain of *D. pseudoobscura* (Richards *et al.* 2005) v2.9 was also included in analyses. The *D. pseudoobscura* strains represent nearly isogenic lines generated by full-sibling matings over several generations (Machado *et al.* 2002; McGaugh *et al.* 2012). Population structure is very limited in *D. pseudoobscura* (Schaeffer and Miller 1992; Noor *et al.* 2000), making it unlikely that the choice of strains, including the reference strain, would influence patterns of diversity. Of the 55 possible pairwise contrasts, 10 of the 11 that showed the lowest pairwise difference at synonymous sites included the reference strain. The reference strain also contributes the smallest number of derived singletons to the polymorphism data set, suggesting that, if anything, there are fewer sequencing errors in the reference strain than in the others.

### Genes

Genes were excluded from analysis if any of the following criteria were met in the reference sequence: the annotated start codon was not AUG; the annotated stop codon was not UGA, UAG, or UAA; any amino acid codon was incompletely resolved; there was a premature stop codon; or the intron/exon boundaries were noncanonical. A total of 3548 genes met all inclusion criteria.

### Recombination rates

**Figure 1:**
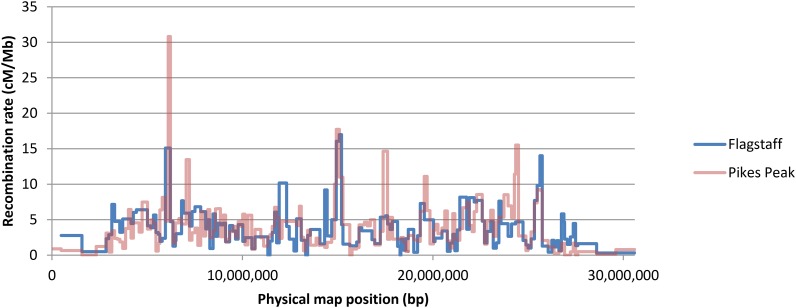
Recombination rate along Chromosome 2. Estimates are from independent testcrosses reported in McGaugh *et al.* (2012).

Estimates were obtained from a pair of testcrosses (Flagstaff and Pikes Peak) described in McGaugh *et al.* (2012). The Flagstaff testcross involved two strains from Arizona bearing the “Arrowhead” arrangement on chromosome 3; the Pikes Peak testcross involved two lines from New Mexico bearing the “Pikes Peak” arrangement on chromosome 3. As these authors noted, recombination rates estimated from the independent testcrosses were very similar along Chromosome 2 (Figure 1). Estimates were obtained for 140 segments in the Flagstaff cross and for 158 segments in the Pikes Peak cross. Except near the ends of the chromosome, most positions are represented in both recombination maps. Unless otherwise stated, for all analyses, the average of the two recombination rates was used for these positions.

### Site inclusion criteria

For each sequence, excluding the reference strain, a base was considered unresolved (’N’) if either the phred consensus quality score was below 30 or the depth of coverage in the alignment was below 15 × . To be included in the analysis, an intron site or complete codon (*i.e.*, all three sites) had to be completely resolved in all 12 strains. A total of 35,376 codons meeting these criteria displayed synonymous polymorphism in *D. pseudoobscura*. Of these, 33,755 segregated two character states; 26 of these loci were in regions for which no recombination rate has been obtained (McGaugh *et al.* 2012) and were excluded from analyses involving recombination. A total of 68,332 intron sites meeting the inclusion criteria were polymorphic in *D. pseudoobscura*; of these, 65,266 segregated two character states.

### Statistical analyses

Statistical analyses were performed using R v3.0.1 for Mac OS X (the R Foundation for Statistical Computing), implemented in RStudio v0.97.551 (RStudio 2013).

### Inference of preferred codons

Following Hey and Kliman (2002), preferred codons were inferred by factor analysis on the 59 codons that encode the 18 degenerate amino acids. Only genes that used all 18 amino acids were included in the factor analysis. The primary factor was polarized, such that values correlated positively with Chi/L (Shields *et al.* 1988) and negatively with effective number of codons (Wright 1990); both are measures of uneven codon usage, with Chi/L increasing and ENC decreasing as codon usage becomes less even. Codons that loaded positively on the primary factor were considered “preferred.” The degree of preference (or “preference score”) of each codon was defined as its loading score (Llopart *et al.* 2008).

For each synonymous polymorphic site, Δpref was defined as pref_derived_ − pref_ancestral_, where pref_derived_ and pref_ancestral_ are the codon preference scores for the derived and ancestral states, respectively, as inferred by parsimony (Llopart *et al.* 2008). For convenience, a site is defined as P→U if Δpref is negative, and as U→P if Δpref is positive. This designation is not clear-cut for amino acids with degeneracy above 2; for example, a mutation may substitute one preferred codon with another preferred codon. However, for our analyses, the polarity of the fitness effect is more important than the assignment of “preferred” or “unpreferred.”

### Analysis of diversity

SFS of derived preferred *vs.* derived unpreferred codons (or similar contrasts) were compared using parametric and nonparametric tests (Akashi and Schaeffer 1997), as well as a permutation test (Llopart *et al.* 2008; described in context under *Site frequency spectra relative to Δpref*).

## Results

### Inference of preferred codons

All C-ending codons, and all but three G-ending codons, are preferred in *D. pseudoobscura*. All A-and T-ending codons are unpreferred (Table 1).

**Table 1:** Codon preference scores

Codon	Amino Acid	Pref Score	Codon	Amino Acid	Pref Score
GCC	ala	0.571	CTG	leu	0.697
GCG	ala	0.076	CTC	leu	0.316
GCT	ala	−0.401	TTG	leu	−0.434
GCA	ala	−0.491	CTA	leu	−0.443
CGC	arg	0.501	CTT	leu	−0.458
CGG	arg	0.163	TTA	leu	−0.572
CGT	arg	−0.090	AAG	lys	0.700
AGG	arg	−0.188	AAA	lys	−0.700
CGA	arg	−0.279	TTC	phe	0.534
AGA	arg	−0.471	TTT	phe	−0.534
AAC	asn	0.461	CCC	pro	0.425
AAT	asn	−0.461	CCG	pro	0.211
GAC	asp	0.392	CCT	pro	−0.404
GAT	asp	−0.392	CCA	pro	−0.442
TGC	cys	0.304	TCC	ser	0.287
TGT	cys	−0.304	AGC	ser	0.270
CAG	gln	0.656	TCG	ser	0.228
CAA	gln	−0.656	AGT	ser	−0.297
GAG	glu	0.724	TCT	ser	−0.351
GAA	glu	−0.724	TCA	ser	−0.456
GGC	gly	0.430	ACC	thr	0.435
GGG	gly	−0.083	ACG	thr	0.204
GGT	gly	−0.222	ACT	thr	−0.357
GGA	gly	−0.291	ACA	thr	−0.416
CAC	his	0.331	TAC	tyr	0.421
CAT	his	−0.331	TAT	tyr	−0.421
ATC	ile	0.584	GTG	val	0.453
ATT	ile	−0.345	GTC	val	0.245
ATA	ile	−0.405	GTA	val	−0.489
			GTT	val	−0.492

Pref, preference.

### Estimates of diversity, divergence, and codon bias

Synonymous sites were counted taking into account the degeneracy of codons. For example, a fourfold degenerate codon would provide one synonymous site, whereas a twofold degenerate codon would provide 1/3 of a synonymous site. A total of 543,985 synonymous sites and 2,746,629 intron sites were completely resolved in all 12 strains. The Watterson (1975) estimator of synonymous θ in *D. pseudoobscura* was 0.0222 across all sites; average synonymous divergence from *D. lowei* was 0.0760. Diversity and divergence varied among amino acids (Table 2), with twofold degenerate amino acids having higher values of both. Watterson’s estimator of intron θ in *D. pseudoobscura* was 0.0085; divergence from *D. lowei* was 0.0297. The lower values for intron sites probably reflect a larger denominator, as each intron site was counted as one full site.

**Table 2:** Estimates of synonymous divergence and diversity

Amino Acid	*N*_syn_*^a^*	*S*_syn_*^b^*	*S*_syn_ (2)*^c^*	*D*_syn_*^d^*	${\hat{\theta }}_{W}$/bp*^e^*	*D*/bp*^f^*
All	543,985.0	35,376	33,729	41,360	0.022203	0.076032
ala	50,380.0	2661	2513	3126	0.018033	0.062048
arg	48,644.0	2558	2365	3047	0.017954	0.062639
asn	13,894.3	1282	1281	1621	0.031502	0.116667
asp	15,130.0	1303	1302	1835	0.029403	0.121282
cys	5,124.6	500	500	576	0.033311	0.112398
glu	18,283.6	1733	1731	1936	0.032361	0.105887
gln	12,420.7	1034	1034	1151	0.028422	0.092668
gly	40,903.0	2600	2427	2932	0.021702	0.071682
his	7051.3	593	593	813	0.028713	0.115298
ile	31,135.4	1833	1782	2028	0.020100	0.065135
leu	91,350.6	5619	5139	6313	0.021001	0.069107
lys	17,437.3	1500	1500	1817	0.029370	0.104202
phe	11,413.0	1358	1356	1634	0.040624	0.143170
pro	35,924.0	2137	1975	2481	0.020310	0.069062
ser	44,903.3	2865	2735	3331	0.021784	0.074182
thr	43,553.0	2406	2257	2775	0.018861	0.063715
tyr	9123.6	851	849	1062	0.031845	0.116401
val	47,313.0	2543	2390	2882	0.018351	0.060913

a Number of synonymous sites in *D. pseudoobscura*.

b Number of synonymous polymorphic sites in *D. pseudoobscura*.

c Number of synonymous polymorphic sites segregating two codons in *D. pseudoobscura*, and for which a recombination rate estimate is available.

d Number of divergent synonymous sites between the reference strain of *D. pseudoobscura* v2.9 (Richards *et al.* 2005) and *D. lowei* for codons fully resolved in all *D. pseudoobscura* strains and in *D. lowei*.

e Watterson (1975) estimator of synonymous theta in *D. pseudoobscura*.

f Synonymous divergence per base pair.

### Association of diversity and codon bias with recombination rate

**Figure 2:**
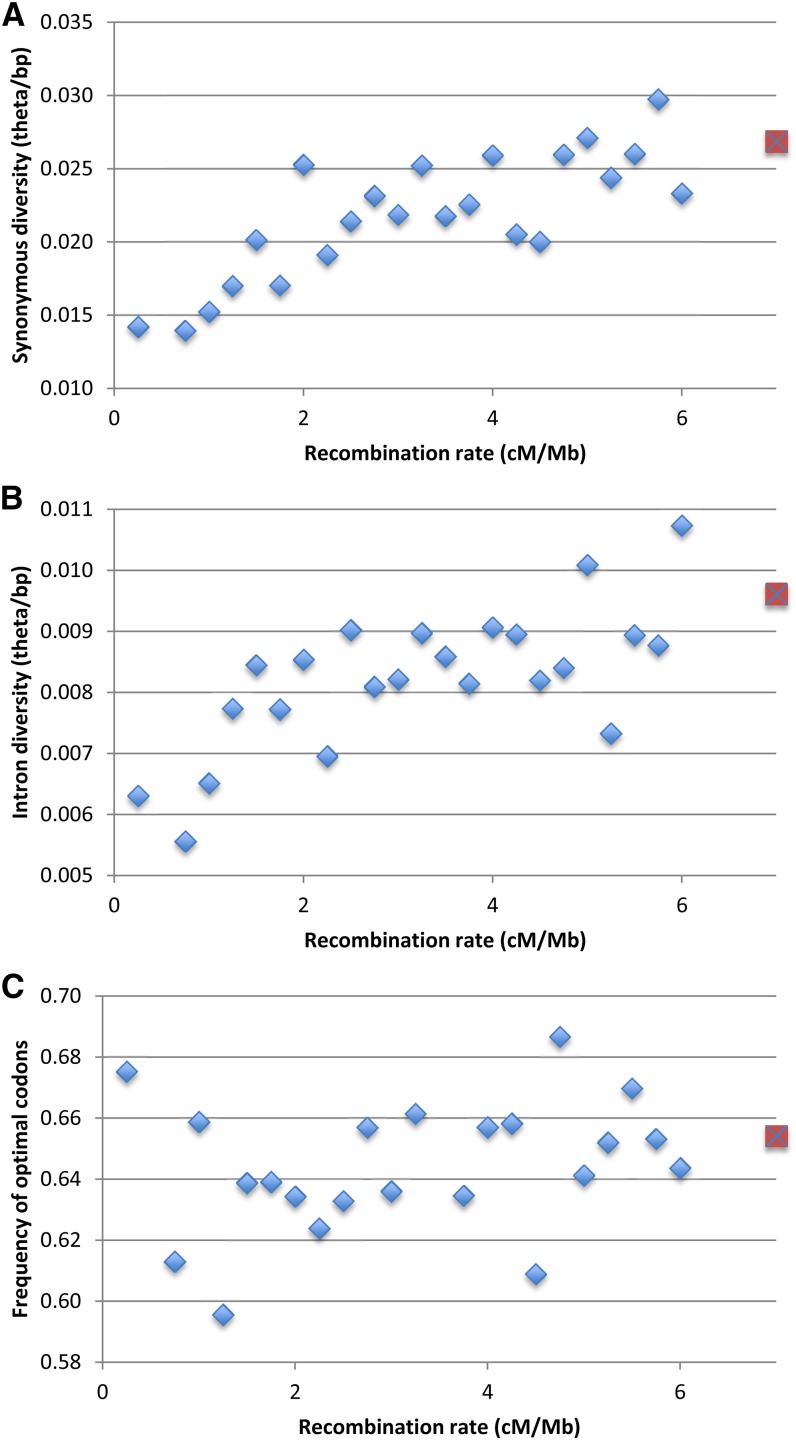
Diversity and codon bias relative to recombination rate. Points are plotted at the upper end of the recombination rate range (*e.g.*, at 0.25 for 0−0.25 cM/Mb); the red point represents sites in regions with recombination rate above 6 cM/Mb. (A) Synonymous diversity measure is the Watterson (1975) estimator of θ. (B) Intron diversity. (C) Codon bias measure is F_op_ (Sharp and Devine 1989).

Using average recombination rate (Flagstaff and Pikes Peak), loci were placed into 25 recombination categories: 0.00−0.25 cM/Mb, >0.25−0.50 cm/Mb, ..., >5.75−6.00 cM/Mb, and >6.00 cM/Mb. (No sites are in regions with >0.25−0.50 cM/Mb.) As shown in Figure 2A, synonymous diversity increases with recombination rate (defined by the upper bound of the category) until about 2 cM/Mb, at which point diversity levels off [*r* = 0.800, 21 degrees of freedom (d.f.), 1-tailed *P* < 10^−5^]. A very similar relationship is observed for intron diversity (*r* = 0.703, 21 d.f., 1-tailed *P* < 10^−4^) (Figure 2B). However, there is no clear relationship between recombination rate and the frequency of optimal codons [F_op_, a measure of preferred codon usage (Sharp and Devine 1989)] (*r* = 0.209, 21 d.f., 1-tailed *P* = 0.158) (Figure 2C).

**Figure 3:**
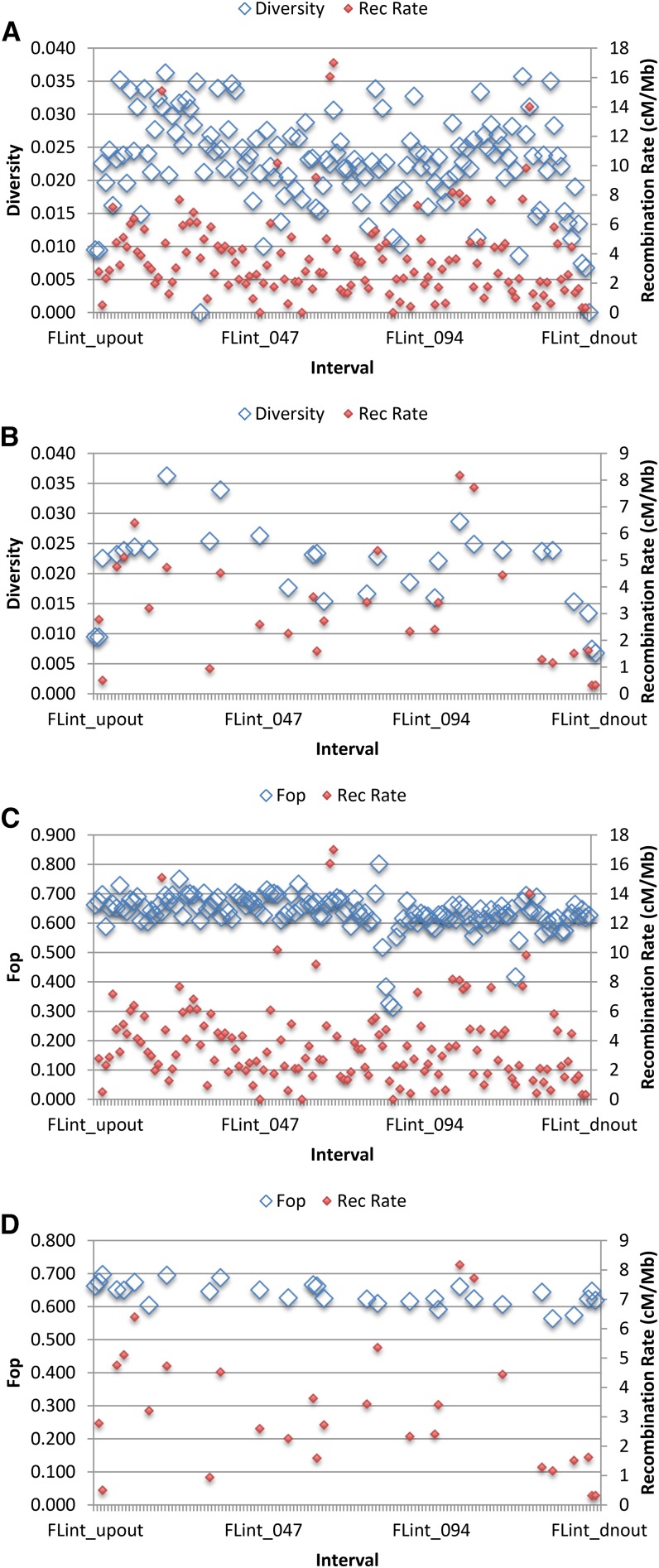
Diversity and codon bias along Chromosome 2. (A) Diversity in all recombination map segments. Segments upstream (FLint_upout) and downstream (FLint_dnout) of the recombination map are also shown; for these, there is no corresponding recombination rate estimate. (B) Diversity in segments with at least 5000 synonymous sites. (C) Codon bias (F_op_) in all segments. (D) Codon bias in segments with at least 5,000 synonymous sites. Recombination rates (cM/Mb from the Flagstaff testcross) are shown for reference.

Synonymous diversity varied spatially along Chromosome 2. Using the 142 segments defined by the Flagstaff recombination map (the 140 regions with recombination rate estimates, along with the two external regions), there is obvious heterogeneity in levels of synonymous diversity (Figure 3A). However, there is also considerable variation in the number of sites analyzed, with as few as 14 sites to as many as 22,635. With a median of 2897 sites per segment, 18 of the 142 segments had fewer than 1000 sites, and 29 had more than 5000 sites. When only the latter are plotted to minimize sampling error (Figure 3B), diversity is clearly reduced at the two ends of the chromosome, and it is also somewhat suppressed in the center of the chromosome. As expected based on the analysis of recombination rate classes, there is a positive correlation between recombination rate in each segment and synonymous diversity (*r* = 0.533, 2-tailed *P* = 0.0026 for the regions with at least 5000 sites). The correlation, although slightly weaker (*r* = 0.350), remains highly significant (*P* < 10^−4^) when all regions are included. For intron sites, the correlation between diversity and recombination rate is somewhat weaker, but still significant, for regions with at least 5,000 sites (*r* = 0.268, *n* = 120, *P* = 0.0029). However, when regions with fewer sites are included, the correlation is lost (*r* = −0.029). In contrast to diversity, variation along Chromosome 2 in F_op_ appears to be negligible (Figure 3, C and D). Results were qualitatively similar, including the significant correlation between recombination rate and either synonymous or intron diversity, using the Pikes Peak recombination map (data not shown).

### Site frequency spectra relative to Δpref

Under a constant-*N*_e_ Wright-Fisher neutral model, the relative frequency of sites with *d*-derived states is 1/*d*, such that the expected mean *d* is (*k* − 1)/*a*, where *k* is the sample size and *a* is the sum of 1, 1/2, ..., 1/(*k* − 1) (Fu 1995). For *k* = 11 *D. pseudoobscura* sequences, we expect 3.414 derived states/site. Overall, our data for synonymous polymorphic sites indicate a shift of the SFS toward lower values, with a mean of 2.59 derived states/site. For intron sites, the mean was 2.279 derived states/site. However, there are noticeably raised tails in the SFSs, likely due in part to ancestral state misassignment (ASM; discussed in *Impact of ASM*).

**Figure 4:**
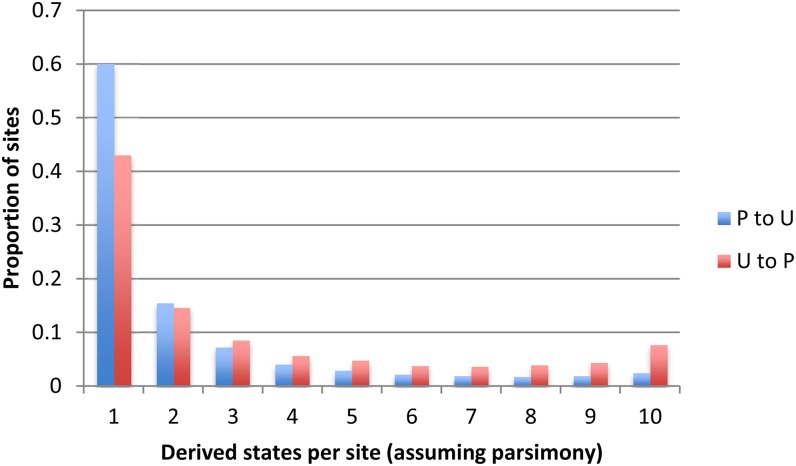
Sites frequency spectrum for synonymous polymorphic sites. Shown are sites that segregate two codons and fall within a region for which recombination rate was estimated. “P to U,” a change to a more unpreferred codon; “U to P,” a change to a more preferred codon.

If natural selection is acting on codon usage, then the SFSs for P→U and U→P changes should be shifted relative to each other; *i.e.*, the SFS for U→P changes should be shifted toward higher values (Akashi and Schaeffer 1997). Consistent with natural selection, the synonymous SFS was shifted toward higher values for the 9918 U→P sites (mean = 3.395) than for the 23,811 P→U sites (mean = 2.258) (Figure 4). However, the SFS for the U→P sites is not right-shifted relative to the expectations of a constant-*N*_e_ Wright-Fisher neutral model; this may indicate a demographic effect on diversity, such as historically increasing *N*_e_ (Tajima 1989a). The difference between U→P and P→U sites is highly significant using either a 1-tailed Student’s *t*-test (t.test in R, *t* = 33.715, *P* < 10^−15^) or a 1-tailed Mann-Whitney *U*-test (following Akashi and Schaeffer (1997)) (wilcox.test in R, *P* < 10^−15^).

Given that singletons (*i.e.*, sites with 1 or 10 individuals carrying the inferred derived state) are the most likely polymorphic sites to represent sequencing errors, the analyses were repeated excluding 5045 U→P and 14,912 P→U singletons. The SFS shift remained highly significant (U→P mean = 4.456; P→U mean = 3.767; *t* = 16.807, *P* < 10^−15^; *U*-test, *P* < 10^−15^). Under a constant-*N*_e_ Wright-Fisher neutral model, the expected frequency of derived states per nonsingleton site would be 4.374. Thus, the SFS for U→P sites was shifted slightly toward higher values, whereas that for P→U sites was shifted toward lower values.

Analyses were repeated for each amino acid. In every case, the SFS was shifted toward higher values for U→P sites than for P→U sites, although there was variation among amino acids in the extent of the shift (see *Variation among amino acids in the SFS shift*). The results were qualitatively unchanged when singletons were excluded (Table 3).

**Table 3:** Shifts in site frequency spectra for each amino acid

Amino Acid	All Sites						Singletons Excluded					
	N U→P*^a^*	N P→U	Mean U→P*^b^*	Mean P→U	*P*-Value *t*-Test*^c^*	*P*-Value *U*-Test*^c^*	N U→P	N P→U	Mean U→P	Mean P→U	*P*-Value *t*-test	*P*-Value *U*-Test
ala	743	1770	3.355	2.267	<10^−15^	<10^−15^	346	682	4.497	3.748	3.43 × 10^−7^	2.54 × 10^−8^
arg	738	1627	2.976	2.410	5.78 × 10^−7^	2.96 × 10^−8^	358	651	4.168	3.902	0.0354	0.0177
asn	553	728	3.562	2.404	4.68 × 10^−13^	4.34 × 10^−14^	279	277	4.466	3.910	0.00246	0.00178
asp	618	684	3.565	2.367	1.39 × 10^−14^	3.02 × 10^−14^	305	259	4.662	3.811	8.26 × 10^−6^	6.95 × 10^−7^
cys	162	338	3.525	2.346	1.43 × 10^−5^	4.278 × 10^−6^	81	125	4.716	3.776	0.00319	8.19 × 10^−4^
gln	232	802	3.608	2.170	1.72 × 10^−10^	1.84 × 10^−10^	112	304	4.875	3.612	1.66 × 10^−6^	2.457 × 10^−7^
glu	408	1323	3.733	2.225	<10^−15^	<10^−15^	198	467	4.768	3.777	1.20 × 10^−6^	3.003 × 10^−6^
gly	850	1577	3.029	2.373	2.47 × 10^−9^	4.26 × 10^−12^	409	597	4.117	3.858	0.0315	0.00371
his	255	338	3.333	1.976	6.14 × 10^−10^	1.08 × 10^−10^	124	108	4.274	3.472	0.00281	0.00347
ile	502	1280	3.171	2.221	2.46 × 10^−11^	5.43 × 10^−12^	243	476	4.412	3.754	1.40 × 10^−4^	2.48 × 10^−4^
leu	1151	3988	3.581	2.217	<10^−15^	<10^−15^	578	1452	4.490	3.766	3.58 × 10^−10^	1.56 × 10^−10^
lys	335	1165	3.878	2.039	<10^−15^	<10^−15^	170	411	4.659	3.640	2.13 × 10^−6^	1.14 × 10^−5^
phe	372	984	4.024	2.181	<10^−15^	<10^−15^	183	360	4.787	3.728	9.97 × 10^−7^	1.42 × 10^−6^
pro	622	1353	3.238	2.362	3.25 × 10^−11^	4.14 × 10^−11^	304	547	4.365	3.826	4.84 × 10^−4^	4.47 × 10^−4^
ser	799	1936	3.299	2.246	<10^−15^	<10^−15^	384	694	4.331	3.710	1.164 × 10^−5^	2.66 × 10^−5^
thr	691	1566	3.148	2.317	8.74 × 10^−12^	3.81 × 10^−12^	340	615	4.412	3.784	2.553 × 10^−5^	1.78 × 10^−4^
tyr	338	511	3.781	2.160	<10^−15^	<10^−15^	195	181	4.482	3.630	8.016 × 10^−5^	4.88 × 10^−4^
val	549	1841	3.353	2.223	<10^−15^	<10^−15^	264	693	4.598	3.755	6.314 × 10^−7^	9.97 × 10^−8^

a N, number of polymorphic sites.

b Mean frequency of derived states/site.

c *P*-values are for 1-tailed tests.

Llopart *et al.* (2008) proposed an alternative test, based on the prediction that natural selection should lead to a positive relationship between *d* and Δpref. Computationally, the sum of *d* × Δpref can serve as a proxy for a correlation or regression coefficient; therefore, 1-tailed statistical significance can be estimated from the proportion of random permutations of *d* *vs.* Δpref that lead to a higher sum of products. The test is significant for all amino acids (including or excluding singletons; see Table 4), as well as for all sites pooled.

**Table 4:** Shifts in site frequency spectra for each amino acid, Monte Carlo analyses*^a^*

Amino Acid	*P*-Value,*^b^* All Sites, Parsimony	*P*-Value, All Sites, Bayesian	*P*-Value, No Singletons, Parsimony	*P*-Value, No Singletons, Bayesian
ala	0*^c^*	0	0	0
arg	0	0	0.02667	0.01952
asn	0	0	0.00238	0.00542
asp	0	0	0.00001	0.00002
cys	0.00002	0.00008	0.00273	0.00527
gln	0	0	0	0
glu	0	0	0	0
gly	0	0	0.02055	0.01272
his	0	0	0.00337	0.00969
ile	0	0	0.00001	0.00002
leu	0	0	0	0
lys	0	0	0	0
phe	0	0	0	0
pro	0	0	0.00009	0.00008
ser	0	0	0	0
thr	0	0	0	0.00001
tyr	0	0	0.00007	0.00067
val	0	0	0	0

a Permutation test of Llopart *et al.* (2008).

b All *P*-values are for 1-tailed tests.

c A reported estimate of 0 indicates that none of 100,000 data permutations led to a higher value of the test statistic.

Llopart *et al.* (2008) also proposed a modification to the test to correct for ASM. Essentially, a simple Bayesian approach was suggested to calculate a posterior odds ratio of correct assignment by parsimony to ASM. The likelihood ratio was based on the neutral SFS in a constant-N population: likelihood ratio = (*k* − *d*)/*d*, where *d* is the frequency of derived states assuming parsimony, *k* is the number of individuals in the sample (here, 11), and *k* − *d* is the number of derived states when parsimony is incorrect (*i.e.*, when there is ASM). The prior odds ratio is the relative probability of no mutation on the branch connecting the outgroup to the base of the ingroup coalescent to the probability of a single mutation on that branch:

$$Prior\;odds\;ratio={\left(\hat{D}-\frac{k-1}{k}\hat{\theta }\right)}^{-1}\;(1),$$ (1)

where $\hat{D}$ and $\hat{\theta }$ are estimates of divergence and diversity, respectively. Thus, following Llopart *et al.* (2008), the posterior odds ratio is calculated as:

$$Posterior\;odds\;ratio=\frac{k-d}{d}{\left(\hat{D}-\frac{k-1}{k}\hat{\theta }\right)}^{-1}(2).$$ (2)

The permutation test can then be performed after randomly assigning an ancestral state for each site using the posterior odds ratio. As shown in Table 4, this usually slightly more conservative test (using distinct estimates of θ and *D* for each amino acid) remains significant for all analyses.

Following up on the recombination analyses, we compared the slopes of the regression lines (*d* on Δpref) for sites in four recombination classes [following McGaugh *et al.* (2012)]: 0−0.5, >0.5−3.0, >3.0−6.0, and >6.0 cM/Mb. For the individual testcrosses (Flagstaff and Pikes Peak), we compared sites in 0-recombination regions to sites found elsewhere. The slope was not lower for the 0−0.5 cM/Mb class (*b* = 0.864, *n* = 1164) than for the three other classes (>0.5−3.0 cM/Mb: *b* = 0.648, *n* = 14,088; >3.0−6.0 cM/Mb, *b* = 0.672, *n* = 13,817; >6.0 cM/Mb, *b* = 0.832, *n* = 4660). For the Flagstaff testcross, the slope was significantly lower for the 0-recombination regions (*b* = 0.164, *n* = 217) than for higher-recombination regions (*b* = 0.694, *n* = 33,226; *P* = 0.0002, 1-tailed Tukey-Kramer test). This result was not mirrored, however, for the Pikes Peak testcross (0-recombination regions: *b* = 0.903, *n* = 1467; higher recombination regions: *b* = 0.679, *n* = 32,262). In fact, mean *d* for U→P changes exceeded mean *d* for P→U changes in all five regions with recombination estimates of 0, the difference ranging from 0.804 (interval 152, *n* = 258) to 2.011 (interval 151, *n* = 133). One-tailed Mann-Whitney *U*-tests were significant after sequential Bonferroni correction (Rice 1989) for four of the five contrasts. The permutation test on each of the five regions produced similar results; all five tests were significant after Bonferroni correction assuming parsimony, and three were significant after Bonferroni correction when allowing for ASM. Therefore, if the slope of the regression line corresponds to effectiveness of selection on codon usage, there is only equivocal evidence for an effect of low recombination in *D. pseudoobscura*.

### Variation among amino acids in the SFS shift

As noted previously, for all amino acids, the average frequency of derived states at synonymous polymorphic sites was greater for P→U changes than for U→P changes. This result is consistent with natural selection on synonymous codon usage. However, it is also consistent with the G/C-biased gene conversion (although recent work by Comeron *et al.* (2012) suggests that that this does not occur in *D. melanogaster*). In the latter, individuals heterozygous for a preferred and an unpreferred codon will usually be segregating a pair of purines or a pair of pyrimidines at the synonymous site (usually the third position of a codon). If heteroduplex intermediates generated during crossing-over tend to resolve toward G or C, then this process could lead to shifts in the SFS even if crossing-over is not, itself, mutagenic.

In the standard genetic code, there are 16 T/C-ending synonymous codon pairs and 13 A/G-ending synonymous codon pairs. Although the degree of bias varies, C- or G-ending codons are usually used disproportionately (Table 5). The C-ending codon is always used disproportionately, although barely so for Asp (50.5% C-ending). For the A/G pairs, the G-ending codon is used disproportionately in all cases except for Gly, where unpreferred GGG is used less often than unpreferred GGA.

**Table 5:** Analysis of variance (site type **×** direction)

Base Change	Effect	d.f.	SS	MS	*F*	*P*-Value
C↔T	Site type*^a^*	1	667	667	107.87	<10^−15^
	Direction*^b^*	1	7556	7556	1,222.32	<10^−15^
	Interaction	1	467	467	75.62	<10^−15^
	Residual	30,844	190,669	6.2		
G↔A	Site type	1	179	179	29.89	<10^−7^
	Direction	1	6829	6829	1,137.35	<10^−15^
	Interaction	1	282	282	46.95	<10^−11^
	Residual	25,420	152,623	6.0		
C↔A	Site type	1	200	200	37.89	<10^−9^
	Direction	1	1235	1235	234.06	<10^−15^
	Interaction	1	97	97	18.31	<10^−4^
	Residual	11,121	58,699	5.3		
G↔T	Site type	1	181	181	34.05	<10^−8^
	Direction	1	1134	1134	212.96	<10^−15^
	Interaction	1	48	48	9.00	0.00271
	Residual	9.848	52,445	5.3		
C↔G	Site type	1	292	292	46.46	<10^−11^
	Direction	1	0.00	0.00	0.00	0.993
	Interaction	1	5.91	5.91	0.94	0.332
	Residual	9,091	57,100	6.3		
A↔T	Site type	1	330.8	330.8	62.78	<10^−14^
	Direction	1	2.2	2.2	0.42	0.519
	Interaction	1	11.9	11.9	2.26	0.133
	Residual	10,981	57,867	5.3		

d.f., degrees of freedom; SS, sum of squares; MS, mean squares.

a Site type can be intron or codon third position.

b Direction can be, for example, C→T or T→C.

**Figure 5:**
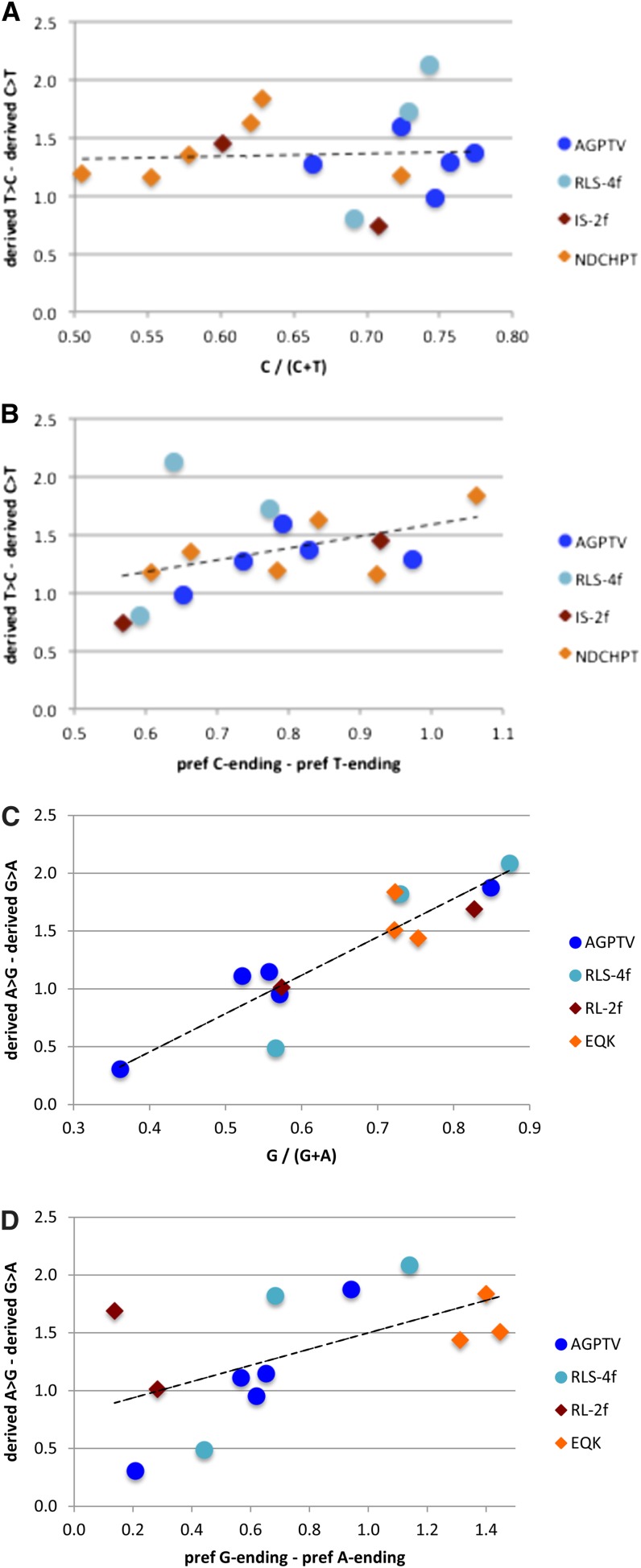
Shifts in site frequency spectra among codon pairs. (A) Difference in average frequency of derived states/polymorphic site for C/T codon pairs relative to codon usage (*i.e.*, proportion of C-ending codons). (B) Difference in average frequency of derived states/site for C/T codon pairs relative to Δpref for T→C changes. (C, D) Corresponding figures for G/A codon pairs. Letters in legend correspond to single-letter amino acid codes; blue, fourfold degenerate amino acids; light blue, codon pair from fourfold degenerate subclass of sixfold degenerate amino acids; red, codon pair from isoleucine or twofold degenerate subclass of sixfold degenerate amino acids; gold, twofold degenerate amino acids. Dashed lines correspond to linear regression through all points.

For all 29 pairs, the average frequency of derived states per polymorphic site is higher for T→C or A→G sites than for corresponding C→T or G→A sites. If biased gene conversion is responsible for these SFS shifts, the magnitude of the shifts should be similar for all codon pairs (at least within the A/G or C/T class). There is, however, considerable variation among codon pairs in relative codon usage and the difference in derived states/site (Table 5). For C/T pairs, two-way analysis of variance [ANOVA; codon pair by direction (C→T *vs.* T→C)] indicated highly significant effects of codon pair (*F*_15,12681_ = 3.859, *P* < 10^−6^) and direction (*F*_1,12681_ = 730.5, *P* < 10^−15^), as well as a highly significant interaction effect (*F*_15,12681_ = 3.250, *P* < 10^−4^). Similar results were obtained for A/G pairs (site type: *F*_12,9514_ = 2.841, *P* < 10^−4^; direction: *F*_1,9514_ = 576.1, *P* < 10^−15^; interaction: *F*_12,9514_ = 5.607, *P* < 10^−8^). For the C/T pairs, the difference in mean *d* correlates moderately with the difference in the preference scores between the C- and T-ending codons, although not well with the degree of bias (Figure 5, A and B). For the A/G pairs, the difference correlates well with degree of bias, and somewhat with the difference in G- and A-ending codon preference scores (Figure 5, C and D).

It is worth contrasting the SFS shifts for codons to those of introns, as shifts in SFSs may provide insight into composition-biasing process, such as biased gene conversion. Using *D. lowei* to infer the ancestral states, the SFSs for all 12 possible changes were obtained. Of particular note are those for C→T, T→C, G→A, and A→G, because these mirror the changes discussed previously for codons. The differences in mean derived states/site, although highly significant, are not as pronounced for introns as they are for synonymous sites. For C→T and T→C, mean *d* was 1.977 and 2.802, respectively (a difference of 0.825). This contrasts with means of 2.231 and 3.582, respectively, for all C/T-segregating codons (a difference of 1.351). Two-way ANOVA on *d* (Table 5) indicated highly significant effects of site type (intron *vs.* codon) and direction, as well as a site type × direction interaction. Similarly, for G→A and A→G intron sites, mean *d* was 1.939 and 2.885, respectively (a difference of 0.946). This contrasts with means of 2.187 and 3.614 for all G/A-segregating codons (a difference of 1.427). Again, two-way ANOVA on *d* indicated highly significant effects of site type and direction, as well as a strong site type × direction interaction. The site type × direction interaction effects indicate significantly larger shifts in codons relative to introns, as expected if the SFS shifts in codons are due to selection, and not only a composition-biasing influence shared by all sites, such as G/C-biased gene conversion.

It is worth noting that restricting the analysis to the much smaller subset of sites in small introns does not qualitatively affect the results. Following Halligan and Keightley (2006), who proposed that short introns were less constrained than longer introns, sites were restricted to introns of 80 bp or shorter, excluding the first nine and last eight bases adjacent to splice junctions. There were 2414 C↔T sites and 1544 G↔A sites. The SFS shift for C↔T sites was slightly reduced (0.645), whereas the SFS shift for G↔A sites (0.935) was essentially unchanged.

Mean *d* in introns was nearly identical for G→C and C→G (2.341 and 2.375, respectively) and for A→T and T→A (2.117 and 2.174, respectively); neither difference was statistically significant. Two-way ANOVA indicated only significant effects of site type (intron *vs.* codon); of note, there was no significant interaction effect (nor was there a significant direction effect). Intermediate results were obtained for G→T *vs.* T→G and C→A *vs.* A→C (both favoring changes toward G or C); all three effects in the ANOVA were significant. The general implication is that the forces shifting SFSs for synonymous codon pairs are stronger than those for introns, consistent with previous observations in Drosophila (De Proce *et al.* 2012). Therefore, although there may be some effect of biased gene conversion on codon usage, this is insufficient to explain our observations.

## Discussion

Our analyses corroborate the likely influence of natural selection on codon usage in *Drosophila*, specifically *D. pseudoobscura*. We also observe, as expected, a positive correlation between diversity and recombination rate. As shown by Kulathinal *et al.* (2008), this association is stronger when recombination is estimated at a finer scale. However, in contrast to analyses on *D. melanogaster*, we observe no significant association of codon bias with recombination rate, despite considerable statistical power and reliable estimates of recombination rate at the finer scale advocated by Kulathinal *et al.* (2008). Evidence from polymorphism data for reduced natural selection on codon usage in areas of low recombination in our dataset is limited at best. It is possible that recombination rate is recently reduced in these areas, such that *N*_e_-reducing effects on diversity of linkage (especially by positive selection on recent beneficial mutations) are apparent, but the *N*_e_ remains sufficient for effective selection. Changes in codon bias would not become apparent for some time, given that this requires accumulation of synonymous substitutions.

Both findings indicate that recombination rate across chromosome 2 exceeds the threshold necessary for effective natural selection. Recombination rates in *D. pseudoobscura* are generally higher than those estimated for *D. melanogaster* (Comeron *et al.* 2012). In *D. melanogaster*, 21% of intervals had recombination rate estimates below 0.5 cM/Mb, in contrast with 6% in *D. pseudoobscura*. Although 59% of intervals had recombination rates above 1.5 cM/Mb in *D. melanogaster* (56% for autosomal regions only), this value was exceeded in 82% of intervals in *D. pseudoobscura*. Furthermore, while the local *N*_e_ could effectively vary across the chromosome due to the *N*_e_-reducing effect of selection on linked sites in areas of lower recombination, the generally higher *N*_e_ in *D. pseudoobscura* may mitigate the Hill-Robertson effect to some extent. That is, the product of *N*_e_ and *s* may be sufficient over most of the chromosome for selection to effectively fix the optimal codons.

### Distortion of the SFS

**Figure 6:**
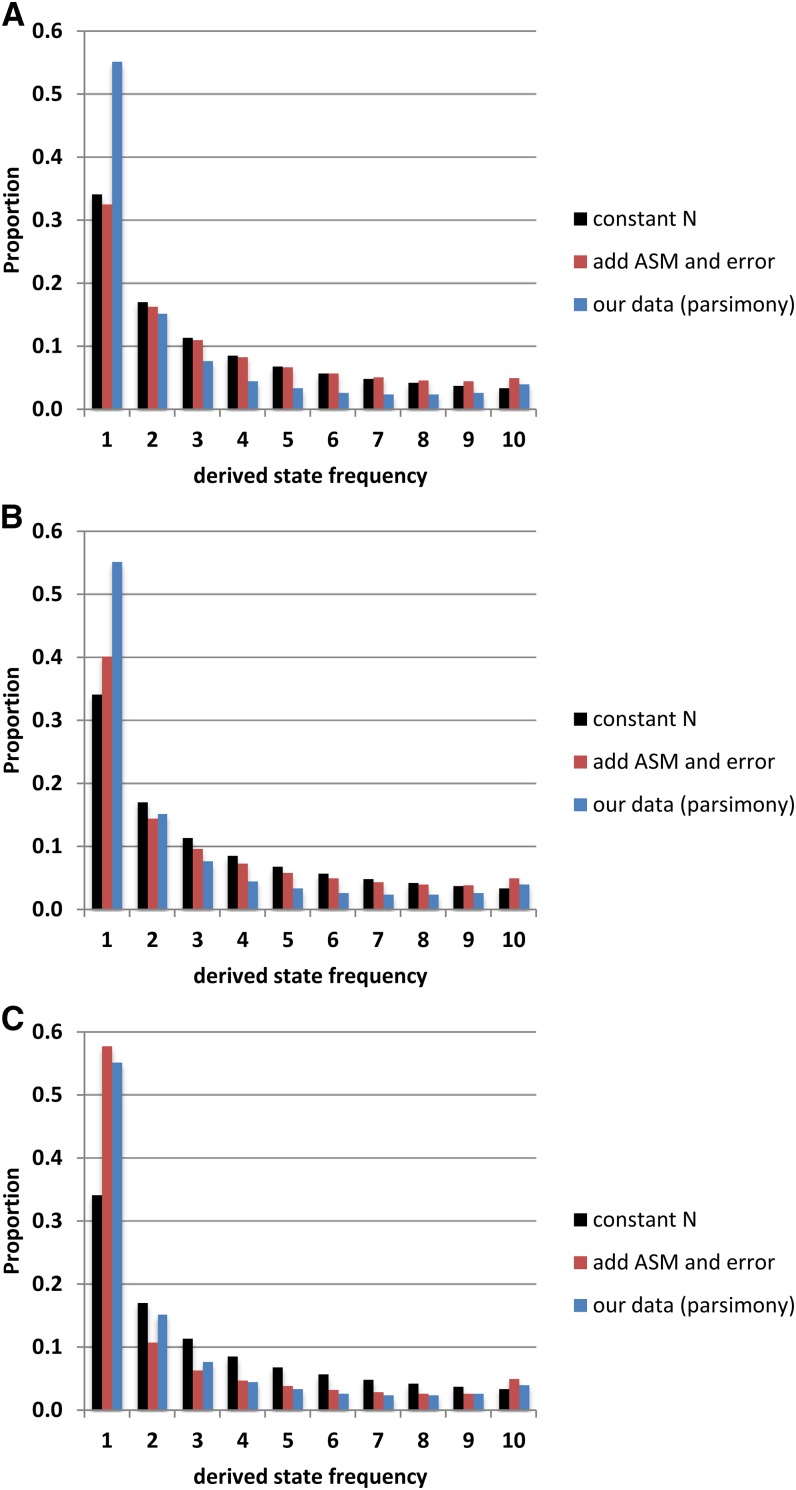
Site frequency spectra corrected for sequencing error and ancestral state misassignment (ASM). Expected proportions under a constant-*N*_e_ Wright-Fisher neutral model are shown in black; our data, assuming parsimony, are shown in blue. (A) Correction for ASM based on observed levels of diversity and divergence (following Llopart *et al.* (2008)). (B) Correction for ASM with a 0.1% sequencing error rate. (C) Correction for ASM with a 0.54% error rate.

The shape of the overall SFS for the 32,729 synonymous polymorphic sites clearly differs from that expected for neutral variants in a constant-N population. First, the proportion of derived singletons (55.1%) is much higher than the 34.1% expected in a population of constant size (Figure 6). Second, there is a raised “tail” in the SFS, with more sites having 10 derived states than 9 derived states.

Biological explanations for an excess of singletons include purifying background selection (Charlesworth *et al.* 1993) and population expansion (Tajima 1989a), both of which distort the coalescent of a population with constant *N*_e_ to increase the relative lengths of branches upon which mutations would be observed as singletons (Tajima 1989a,b). Background selection or a demographic influence on the SFS should affect U→P and P→U sites similarly, but we observe a significantly stronger shift toward low-frequency derived states in P→U sites. Therefore, while both influences may be at work in *D. pseudoobscura*, they are not sufficient to explain our observations.

In addition to demographic effects, an excess of singletons can arise, in principle, from sequencing error. With a low value of θ, most sites in a small sample (here, *k* = 11) would be invariant. A single error at an invariant site would produce an apparent singleton. Additional errors would add to the remainder of the SFS, but assuming that errors are independent, the impact would be seen mainly on singletons. Assuming a 0.1% error rate (equivalent to a phred consensus quality score of 30), following the binomial distribution, 98.91% of truly invariant sites would be observed as invariant. However, 1.09% of invariant sites would be apparent singletons, whereas only 5.5 × 10^−3^% would appear to have two derived states (assuming that all errors produce the same character state). Likewise, 99.00% of true singletons would be observed as singletons, whereas 0.99% would present as having two derived states (*i.e.*, be observed as “doubletons”) and 4.4 × 10^−3^% would present as having three derived states. This approach can be extended to all possible values for true and observed derived states (*i.e.*, for a true invariant site appearing to have 1, 2, ... 10 derived states; for a true singleton appearing to have 2, 3... 10 derived states; and so on), ultimately leading to 42.3% of sites having one apparent derived state. This is well below the observed value of 55.1%, which would require an error rate of approximately 0.33%. Given the requirements of consensus quality scores of 30 or better, a minimum of 15 × coverage in highly inbred strains, and full resolution of all three codon positions in every strain (including the outgroup), sequencing error alone does not explain the high proportion of singletons observed.

It is further unlikely that the resequencing using the Illumina platform is leading to an accumulation of false As and Ts. Across a range of genomic G+C contents, Nakamura *et al.* (2011) found that A/T→G/C errors were more likely than G/C→A/T errors. Although Illumina sequencing is more prone to base call errors than either ABI SOLiD or Roche 454 (Liu *et al.* 2012), error bias is unlikely to explain our observed shifts in SFSs. Most A/T→G/C polymorphic sites reflect U→P changes in *D. pseudoobscura* yet the proportion of singletons is much lower for U→P sites (43.1%) than it is for P→U sites (60.7%; Figure 4).

### SFS differences among codon pairs

The SFS data are consistent with an influence of natural selection on codon usage in *D. pseudoobscura*, corroborating previous studies (Akashi and Schaeffer 1997; Haddrill *et al.* 2011). Additionally, the data indicate that intensity of selection varies among synonymous mutations. Vicario *et al.* (2007) previously proposed this possibility in a comparison of codon usage in the genomes of 12 Drosophila species. Although there is a correspondence between codon bias and SFS shifts consistent with differential selection, the nature of that differential selection is uncertain. Selection does not appear to be strongest on the more common amino acids within a degeneracy class, as might be expected for selection on efficiency of translation. For example, phenylalanine and tyrosine are used at an intermediate level among the C/T-ending twofold degenerate amino acids, despite showing the strongest SFS shifts in this class. Alanine is the most commonly used fourfold degenerate amino acid, but its SFS shifts for C↔T and A↔G changes are intermediate within the degeneracy class. On the other hand, for sixfold degenerate amino acids, leucine and serine are used more often than arginine (and are among the most used amino acids overall) and show much stronger SFS shifts for changes in the fourfold degenerate subsets; in fact, their SFS shifts are among the strongest observed in this analysis. Vicario *et al.* (2007) proposed *ad hoc* explanations for stronger selection on some amino acids (*e.g.*, for accurate translation of disulfide bridge-forming cysteine or for accurate and efficient translation of heavily used hydrophobic leucine). Potential influences of isoaccepting tRNA pools on codon bias have been identified for Drosophila (Moriyama and Powell 1997; Powell and Moriyama 1997), but these authors note that pools likely change over time within an individual (White *et al.* 1973) and that this plasticity may itself influence codon bias among amino acids. Furthermore, the complement of tRNA genes likely changes over evolutionary time in Drosophila, with evidence of numerous gains, losses, and reassignments (Rogers *et al.* 2010). The observed slightly stronger shifts for A↔G *vs.* C↔T synonymous changes may reflect differences in composition-biasing influences, as reflected in corresponding SFS shifts for introns. It is, therefore, probably premature to speculate too extensively on the bases of differential selection among amino acids.

That this main result is not an artifact of sequencing error is reinforced by differences among codon pairs in the proportion of derived singletons and the proportion of polymorphic sites. Variation in the proportion of derived singletons among all 16 ancestrally C-ending codons is not quite significant (*G* = 24.91, 15 d.f., *P* = 0.0511), and there is no significant variation for ancestral T-ending codons (*G* = 14.85, 15 d.f., *P* = 0.462). However, variation in the proportion of derived singletons is significant for both ancestral G-ending (*G* = 21.42, 12 d.f., *P* = 0.0445) and A-ending codons (*G* = 22.98, 12 d.f., *P* = 0.0279).

For C/T codon pairs, 3.25% of ancestral C-ending codons are polymorphic, whereas 2.51% of ancestral T-ending codons are polymorphic; this difference is highly significant (*G* test of independence, *G* = 189.4, 1 d.f., *P* < 10^−15^). Although this finding could indicate a higher probability of C→T errors (which is not likely with Illumina platforms; see *Distortion of the SFS*), there is considerable variation among C/T codon pairs in the proportion of polymorphic ancestral C-ending codons (*G* test of independence: *G* = 199.3, 15 d.f., *P* < 10^−15^) and polymorphic T-ending codons (*G* = 197.4, 15 d.f., *P* < 10^−15^). Similar results were obtained for G/A codon pairs; 3.13% of ancestral G-ending codons were polymorphic and 2.22% of ancestral A-ending codons were polymorphic (*G* = 224.5, 1 d.f., *P* < 10^−15^) . However, the proportions varied among codon pairs (ancestral G-ending: *G* = 102.2, 12 d.f., *P* < 10^−15^; ancestral A-ending: *G* = 42.7, 12 d.f., *P* < 10^−4^) (see Table 6 for proportions). Examining codon pairs from fourfold degenerate sites only, *G* tests remained significant for all four ancestral bases (all *P*-values below 10^−4^). Likewise, for twofold degenerate sites only, *G* tests were highly significant for ancestral C-ending and T-ending codons (*P* < 10^−15^) and for ancestral G-ending codons (*P* = 0.00012); however, there was no significant heterogeneity among codon pairs for ancestral A-ending codons (*P* = 0.403). Thus, there is considerable variation in the proportion of polymorphic codons, even within similar degeneracy classes, a result that does not support a major role for sequencing error, but is consistent with differences among codon pairs in the influence of weak selection.

**Table 6:** Summary data for C/T and G/A segregating and fixed different codon third positions

Codon Pair	Amino Acid	C_3_ or G_3_*^a^*	Δpref_TA->CG_*^b^*	*S*/*N* (CG)*^c^*	*S*/*N* (TA)*^d^*	${\bar{d}}_{CG->TA}:{\bar{d}}_{TA->CG}$*^e^*
GC C/T	Ala	0.757	0.972	684/24,976	193/8,275	2.251:3.539
GG C/T	Gly	0.747	0.652	574/19,504	226/7,027	2.336:3.319
CC C/T	Pro	0.774	0.829	428/14,444	97/4,144	2.185:3.557
AC C/T	Thr	0.724	0.792	458/15,326	104/6,117	2.216:3.808
GT C/T	Val	0.663	0.737	355/12,959	98/7,011	2.352:3.622
CG C/T	Arg4	0.692	0.591	509/14,398	194/7,044	2.369:3.170
CT C/T	Leu4	0.728	0.774	525/14,629	116/5,869	2.051:3.776
TC C/T	Ser4	0.743	0.638	436/14,149	150/5,164	1.959:4.093
AT C/T	Ile	0.601	0.929	753/21.090	241/15,310	2.112:3.560
AG C/T	Ser2	0.708	0.567	468/16,090	223/7,353	2.348:3.090
AA C/T	Asn	0.552	0.922	728/21,727	553/19,956	2.404:3.562
GA C/T	Asp	0.505	0.784	684/21,184	618/24.206	2.367:3.565
TG C/T	Cys	0.723	0.608	338/10,916	162/4,458	2.346:3.525
CA C/T	His	0.578	0.662	338/11,373	255/9,781	1.976:3.333
TT C/T	Phe	0.629	1.064	984/20,878	372/13,361	2.181:4.024
TA C/T	Tyr	0.621	0.842	511/15,979	338/11,392	2.160:3.781
GC G/A	Ala	0.522	0.567	278/8.438	161/8,691	2.392:3.503
GG G/A	Gly	0.361	0.208	229/4,821	201/9,551	2.655:2.960
CC G/A	Pro	0.557	0.653	308/8,910	181/8,456	2.360:3.508
AC G/A	Thr	0.571	0.620	408/12,025	181/10,085	2.368:3.320
GT G/A	Val	0.849	0.942	631/23,036	113/4,307	2.019:3.894
CG G/A	Arg4	0.566	0.442	217/5,828	115/5,398	2.618:3.104
CT G/A	Leu4	0.874	1.140	959/34,643	169/5,649	2.088:4.172
TC G/A	Ser4	0.730	0.684	399/12,539	102/5,237	2.120:3.941
AG G/A	Arg2	0.574	0.283	145/4,236	70/3,394	2.159:3.171
TT G/A	Leu2	0.827	0.138	334/12,658	74/2,788	2.135:3.824
GA G/A	Glu	0.722	1.448	1323/38,468	408/16,383	2.225:3.733
CA G/A	Gln	0.753	1.312	802/28,876	232/10,386	2.170:3.608
AA G/A	Lys	0.723	1.400	1165/37,359	335/14,953	2.039:3.878

a Usage of the codon ending in C or G for a C/T or G/A codon pair, respectively.

b Δpref for a substitution of a T- or A-ending codon with the corresponding C- or G-ending codon.

c *S*, frequency of polymorphic sites with C or G as the ancestral state; *N*, frequency of sites with C or G as the ancestral state; frequencies are reported only for sites that are fully resolved at all three codon positions in all *D. pseudoobscura* and *D. lowei* sequences.

d *N* and *S* for sites with T or A as the ancestral state (see c).

e Mean frequency of derived states per site; CG→TA, ancestral state ends with either C or G; TA→CG, ancestral state ends with either T or A.

### Impact of ASM

The raised tail of the SFS may be explained by ASM, especially if the SFS is already left-shifted. Under a model with constant *N*_e_, we expect a ratio of 10:9 for sites with 9 *vs.* 10 derived states. However, if we allow ASM with a probability of 1/(LR+1), where LR is the likelihood ratio in Equation 1, we can solve for the value of divergence required for a given value of θ that would lead to a 1:1 ratio by (a) factoring in sites with 1 or 2 derived states that present as having 10 or 9 derived states and (b) factoring out sites with 10 or 9 derived states that would present as having 1 or 2 derived states. For our θ = 0.0222, a divergence of 0.0424 would be sufficient and would lead to 4.1% of sites presenting 9 and 4.1% of sites presenting 10 derived states under a constant-N model. While both values exceed our observations, the excess of singletons by necessity decreases the proportion of sites in the remainder of the remainder of the SFS. We estimated per-site synonymous divergence at 0.0763, which leads to a slightly raised tail (a ratio of 1.12 for 10:9 derived states); we observed a ratio of 1.54.

If we assume a constant-N model, but with ASM and a sequencing error rate of 0.1%, we can begin to approach the observed SFS (Figure 6). However, we still observe an excess of singletons; we still need a much higher error rate to approach the observed SFS. In fact, for P→U sites, the error rate would have to be approximately 0.54% to produce the extreme left shift. It is not possible to reproduce the SFS for U→P sites, with a slight excess of singletons and a markedly raised tail, with sequencing error and ASM alone.

Analysis of SFS of derived synonymous mutations in *D. pseudoobscura* indicates that the intensity of natural selection varies among classes of synonymous mutation. The shapes of the SFSs are likely shaped by other influences, possibly including ASM and sequencing error, but variation among synonymous codon pairs in the extent of the SFS shifts support differential intensity of selection.

## Supplementary Material

Corrigendum
