# Supplementary material for: Evidence that Natural Selection on Codon Usage in Drosophila pseudoobscura Varies Across Codons
Source: G3 (Bethesda). 2014 Feb 13;4(4):681–92. doi: 10.1534/g3.114.010488 (PMC4059240; doi:10.1534/g3.114.010488)
Supplement: Corrigendum [file supp_4_4_681__index.html]

Corrigendum 

# Evidence that Natural Selection on Codon Usage in *Drosophila pseudoobscura* Varies Across Codons

## Corrigendum for Kliman, G3: Genes|Genomes|Genetics 4 (4) 681‐692.

**Files in this Data Supplement:**

- Corrigendum - Corrigendum for Kliman, G3: Genes|Genomes|Genetics 4 (4) 681‐692.
