# Supplementary material for: Evidence that Natural Selection on Codon Usage in Drosophila pseudoobscura Varies Across Codons
Source: G3 (Bethesda). 2014 Feb 13;4(4):681–92. doi: 10.1534/g3.114.010488 (PMC4059240; doi:10.1534/g3.114.010488)
Supplement: Corrigendum [file supp_g3.114.010488_Corrigendum_Kliman.pdf]

Corrigendum for Kliman, *G3* 4 (4) 681-692.

G3, Vol 4, 681-692, April 2014, Copyright © 2014 Genetics Society of America.

CORRIGENDUM

In the article by R. M. Kliman (G3 4: 681-692) entitled "Evidence that Natural Selection on Codon Usage in *Drosophila pseudoobscura* Varies Across Codons", Figure 5 parts C and D have been modified to correct an error in transcribing values during the production of the figure. None of the analyses were affected.
